# Perceived Stress and Psychological Distress in Prosthodontic Residents: A Systematic Review and Critical Appraisal of Evidence Gaps in Occupational Burnout

**DOI:** 10.7759/cureus.105553

**Published:** 2026-03-20

**Authors:** K Anbarasi, Prathibha Saravanakumar, Manigandan Kuzhanchinathan, Manohar Rajkumar, Selvakumar Haridoss, Kavitha Swaminathan

**Affiliations:** 1 Oral Medicine and Radiology, Sri Ramachandra Institute of Higher Education and Research, Chennai, IND; 2 Prosthodontics, Sri Ramachandra Institute of Higher Education and Research, Chennai, IND; 3 Conservative Dentistry and Endodontics, Sri Ramachandra Institute of Higher Education and Research, Chennai, IND; 4 Public Health Dentistry, Sri Ramachandra Institute of Higher Education and Research, Chennai, IND; 5 Pediatric and Preventive Dentistry, Sri Ramachandra Institute of Higher Education and Research, Chennai, IND

**Keywords:** burnout, dental education, good health and well-being, mental health, occupational health, occupational stress, prosthodontics, work-related stress

## Abstract

Stress, psychological distress, and burnout are recognized occupational hazards in dentistry; however, the burden has not been synthesized separately for prosthodontics despite the specialty’s distinctive combination of high-precision rehabilitation, prolonged treatment cycles, aesthetic expectations, laboratory dependency, and repeated adjustments. This systematic review followed PRISMA 2020 guidance and was prospectively registered in the PROSPERO database (CRD420261277566). The review had three objectives: (1) to synthesize perceived stress and psychological distress among prosthodontists and prosthodontic residents, (2) to determine whether validated instruments have been used to measure occupational burnout in this population, and (3) to appraise the methodological quality of the available evidence. Electronic searches were undertaken in PubMed, Scopus, Cochrane Library, LILACS, Google Scholar, and supplementary sources from inception to 10 January 2026. Additional searches included Shodhaganga, targeted web searches, and backward citation tracking. Three cross-sectional studies met the eligibility criteria. Two studies evaluated North American prosthodontic residents, and one evaluated postgraduate prosthodontic trainees in Saudi Arabia. Across studies, stress clustered around academic overload, faculty-related pressures, lack of time for personal life or leisure, inadequate support, and clinical or examination requirements. One North American study reported that more than one-third of residents exceeded the study-defined threshold for high stress, whereas a multicenter US study found that prosthodontics residents showed higher mean stress scores than pediatric dentistry and oral and maxillofacial surgery residents across multiple domains. Importantly, no eligible study used a validated burnout inventory; therefore, prosthodontics-specific burnout prevalence could not be estimated. JBI appraisal showed variable methodological quality, with concerns centering on confounding, sampling, and response rates. Current evidence, therefore, indicates elevated stress among prosthodontic residents, but a major evidence gap remains regarding occupational burnout measured using validated instruments.

## Introduction and background

Stress and burnout are increasingly recognized as occupational hazards across health professions, and dentistry is no exception. Contemporary literature describes a broad spectrum of dentists’ mental health challenges, including stress, burnout, anxiety, depression, and related adverse outcomes, and links these to the unique pressures of clinical practice, patient management, and professional expectations [1]. In prosthodontics, the clinical and administrative context may plausibly intensify occupational strain because prosthodontic practice often involves high procedural complexity, prolonged chairside time, demanding aesthetic expectations, medically compromised/elderly patients, frequent remakes/adjustments, financial pressure, and substantial administrative burden [2].

Existing dentistry-wide evidence indicates a substantial burden of mental health symptoms among dental practitioners [3]. In a large national cross-sectional survey of Australian dental practitioners, self-reported psychological distress was common, and approximately one in four participants were classified as likely experiencing burnout; the study also operationalized psychological distress using validated tools (Kessler Psychological Distress Scale and GHQ-12) and burnout using the Sydney Burnout Measure [4]. UK survey data similarly highlight high levels of psychological distress and burnout-related symptoms among dentists, including emotional exhaustion [2]. In addition to prevalence estimates, qualitative and mixed-methods work emphasizes that dentists’ mental health can be shaped by professional role demands and work conditions (e.g., staffing, autonomy, and practice management), with downstream implications for productivity, attrition, and patient care [5]. Dentistry-specific stressors reported in the literature include patient expectations, time pressures, aesthetic demands, business pressures, staffing problems, and regulatory requirements [4].

Despite that broader dentistry burden, extrapolation to prosthodontics is methodologically limited. Specialist training structure, patient mix, treatment duration, laboratory coordination, remake burden, and performance pressure differ meaningfully from general dental practice. In addition, mixed-specialty dental studies often do not report prosthodontic subgroup data separately. The available indirect comparative evidence is notable: in a recent US multi-program study, prosthodontics residents had higher mean stress scores than pediatric dentistry and oral and maxillofacial surgery residents across several domains, including personal-life stress, faculty-related stress, academic stress, and future-related stress [1]. For these reasons, a prosthodontics-focused synthesis is warranted. The objectives of this review were: (1) primary objective: to synthesize the available evidence on perceived stress and psychological distress among prosthodontists and prosthodontic residents; (2) secondary objective: to determine whether validated burnout instruments have been used to quantify occupational burnout in this population; and (3) exploratory objective: to summarize the instruments used, key stressors reported, and methodological quality of the included studies.

## Review

Materials and methods

Protocol and Registration

This systematic review was conducted in accordance with PRISMA 2020 guidelines and was prospectively registered with PROSPERO (CRD420261277566). 

Eligibility Criteria

Population included prosthodontists, prosthodontic residents, fellows, or postgraduate trainees in any clinical or academic setting. Outcomes included perceived stress, psychological distress, or occupational burnout measured with validated instruments or structured specialty-specific stress surveys. Study designs included observational studies and extractable baseline data from trials. Language was restricted to English. Mixed-population studies were excluded unless prosthodontic subgroup data were extractable and reported separately. Studies that included multiple dental specialties without separable prosthodontic results were excluded.

Information Sources and Search Strategy

Electronic searches were performed in PubMed, Scopus, Cochrane Library, LILACS, and Google Scholar (first 100 results by relevance) from database inception to 10 January 2026. The core search strategy combined prosthodontics-related population terms with outcome terms for stress, psychological distress, and burnout. Full database-specific strategies are provided in Supplementary File 1. Supplementary searching included Shodhaganga, targeted Google web searching, and backward citation checking of included studies. The 10 records identified via other methods in the PRISMA flow diagram all came from Shodhaganga and were retrieved using the keywords “prosthodontics stress,” “prosthodontic residents stress,” “prosthodontics burnout,” and “prosthodontist burnout.”

Study Selection

All records were imported into Rayyan for de-duplication and screening [6]. Title/abstract screening and full-text assessment were performed independently by two reviewers, with disagreements resolved by discussion and, when required, adjudication by a third reviewer. Reasons for exclusion at the full-text stage were recorded.

Data Extraction and Data Items

Two reviewers independently extracted data using a standardized form. Extracted items included author, year, country, setting, participant group, sample size, response rate, instrument used, scoring/threshold definitions, main stress or burnout outcomes, and reported explanatory variables or correlates.

Outcomes

The primary outcome was perceived stress or psychological distress among prosthodontic trainees or specialists. The secondary outcome was occupational burnout measured with validated burnout inventories such as the Maslach Burnout Inventory (MBI), Copenhagen Burnout Inventory (CBI), Oldenburg Burnout Inventory, or equivalent tools [7-11].

Risk of Bias Assessment

Methodological quality was assessed independently by two reviewers using the Joanna Briggs Institute (JBI) critical appraisal checklist for analytical cross-sectional studies. Item-level judgements were recorded for each study [12]. For descriptive summary only, the number and proportion of checklist items rated "Yes" were calculated; these summaries were not used as exclusion criteria.

Data Synthesis

A synthesis without meta-analysis (SWiM)-informed narrative synthesis was undertaken because the studies were few and heterogeneous. Results were grouped first by outcome construct (stress/psychological distress, followed by burnout) and then by instrument and key findings. Random-effects meta-analysis for pooled prevalence had been prespecified for sufficiently comparable outcomes; however, pooling was not undertaken because instruments, scoring systems, thresholds, and subgroup structures differed substantially across the three eligible studies. Missing data were not imputed; studies without extractable prosthodontic subgroup results were excluded.

Results

Study Selection

**Figure 1:**
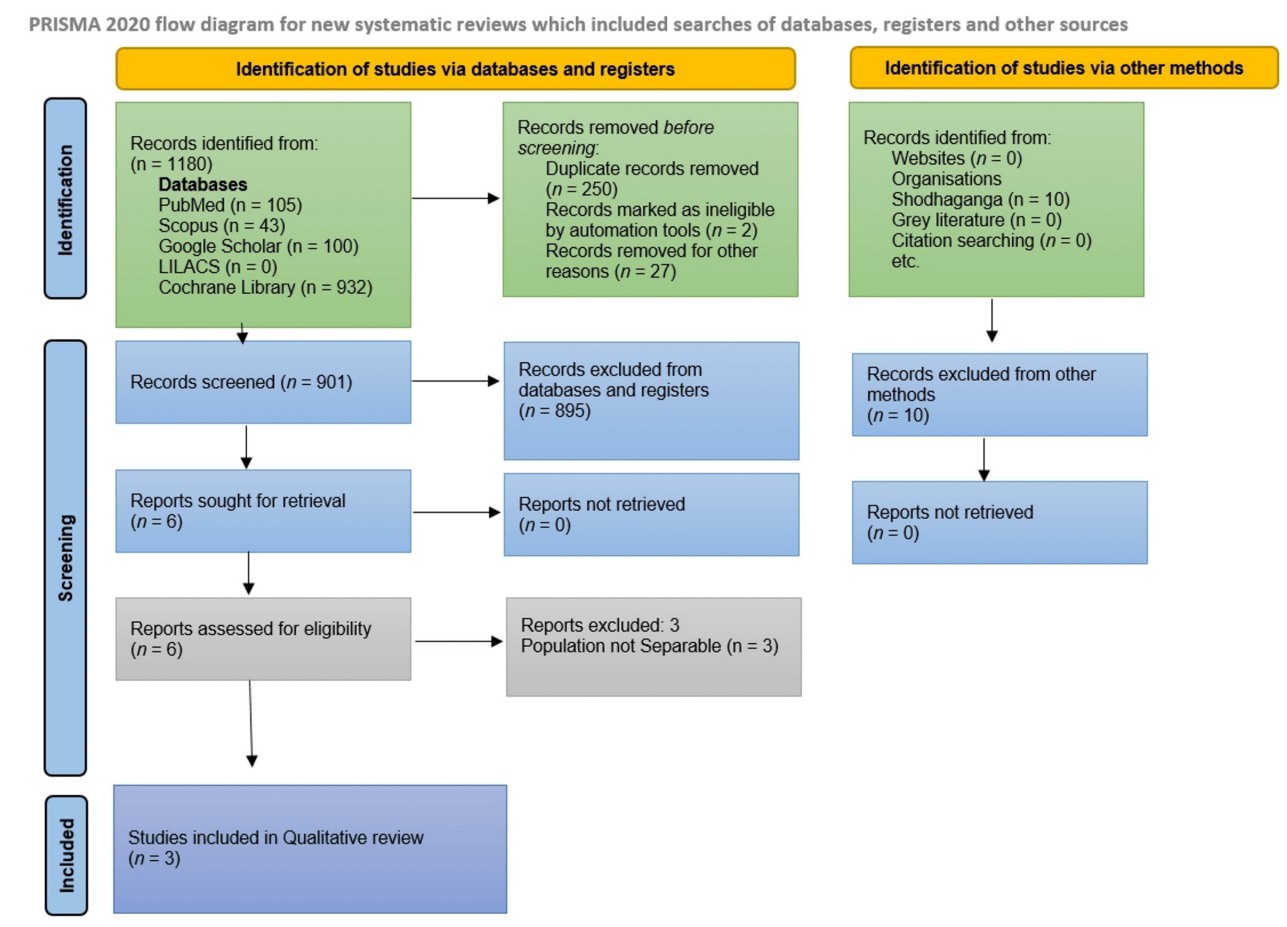
PRISMA 2020 flow diagram of study selection PRISMA: Preferred Reporting Items for Systematic Reviews and Meta-Analyses

Database and register searches yielded 1180 records: PubMed (n=105), Scopus (n=43), Google Scholar (n=100), LILACS (n=0), and Cochrane Library (n=932). An additional 10 records were identified through other methods, all from Shodhaganga. After removal of 250 duplicates, two automation-flagged ineligible records, and 27 records removed for other reasons, 901 records remained for title/abstract screening. Of these, 895 were excluded. Six reports were sought for retrieval; all six were retrieved, and six full texts were assessed. Three full-text reports were excluded because prosthodontic data were not separately extractable, leaving three studies for qualitative synthesis. Excluded full-text reports are listed in Supplementary File 2. The study selection process is summarized in Figure 1.

Characteristics of Included Studies

All three included studies were cross-sectional. Almabadi et al. evaluated postgraduate prosthodontics trainees in a single Saudi institution (n=35/35; all 35 eligible prosthodontics postgraduates were contacted and participated) using a modified Dental Environment Stress Scale tailored to prosthodontics [13]. Punj et al. surveyed prosthodontic residents across American Dental Association (ADA)-accredited North American residency programs (64 responses; 59 analyzable; response rate 13.2%) using a modified Graduate Dental Environment Stress survey and open-ended items [14]. Inglehart et al. surveyed residents across 17 U.S. residency programs; the prosthodontics subgroup comprised 44 of 212 total dental residents, with a prosthodontics response rate of 14% [15]. Table 1 summarizes study characteristics and the JBI appraisal profile.

**Table 1:** Characteristics of included studies, study setting, sample size and response rate, outcome instruments, key prosthodontics-relevant findings, and JBI summary rating GDES: graduate dental environment stress; DES: dental environment stress; ADA: American Dental Association; JBI: Joanna Briggs Institute; OMS: oral and maxillofacial surgery; PD: pediatric dentistry

Author, year, country	Study design	Population	Sample size (n)/response	Outcome instrument(s)	Outcomes	Key findings relevant to prosthodontics	JBI overall rating
Almabadi AA, 2025 (Saudi Arabia) [13]	Cross-sectional survey (pilot/exploratory), single institution	Postgraduate prosthodontics students/residents (Master’s+Board programs), Prosthodontics	n=35 participated; survey distributed to all eligible prosthodontics postgraduates (35/35)	Modified DES Scale tailored to prosthodontics (30 items; Likert: no stress/mild-moderate/severe; some items “not applicable”); internal consistency Cronbach’s α=0.856	Severe stress (domain-level %): academic overload 62.9%, fear of failure 51.4%, completing examination requirements 48.6%, difficulty obtaining suitable patients 48.6%, time limitation 45.7%, difficulty completing class work 42.9%, lack of confidence about being a successful dental resident 42.9%, examination and grading 40.0%. No statistically significant differences in major severe stressors by gender/year/previous qualification after Bonferroni correction (q>0.999). Duration of prior experience associated with fear of failure (q=0.003) and completing examination requirements (q=0.045)	Prosthodontics-specific stressors were prominent: prosthodontic lab support (31.4% severe), patient availability for clinical requirements (48.6% severe), and high academic/curriculum workload pressures. Findings highlight that workload and fear-of-failure dominate stress profiles in prosthodontic postgraduate training and may justify structured stress monitoring and targeted institutional supports (e.g., patient allocation systems and lab support strengthening)	6/8 items met; 1 unclear; 1 not met
Punj et al., 2025 (North America) [14]	Cross-sectional mixed-methods (quantitative survey+open-ended questions)	Postgraduate prosthodontic residents	64 respondents; 59 analyzed (5 excluded: missing all variables). Response rate 13.2% (denominator estimated ≈482 residents in ADA-accredited programs)	Modified GDES; qualitative open-ended items (open coding; NVivo)	High stress defined as GDES ≥76; median total score 69 (IQR 58-84). High-stress prevalence 37% (95% CI 25-51)	Top stressors: lack of time for leisure, lack of adequate staff, neglect of personal life. Coping themes most referenced: self-care, time management, connecting with others	4/8 items met; 4 unclear; 0 not met
Inglehart et al., 2024 (USA) [15]	Cross-sectional survey (comparative across programs)	Dental residents: pediatric dentistry, prosthodontics, OMS	Total 212: PD 112, Prosthodontics 44, OMS 56. Response rate: PD 12.6%, prosthodontics 14%; OMS response rate not calculable	Revised graduate student stress scale (28 items; factor-derived indices). Also assessed harassment/discrimination and satisfaction domains	Stress reported as mean index scores on a 4-point scale (factor-derived indices: personal life-related, faculty-related, lack of confidence, academic, patient-related, future-related stress)	Prosthodontics residents showed highest stress across several domains (e.g., personal life-related stress) and lowest job satisfaction among the three specialties	2/8 items met; 4 unclear; 2 not met

Perceived Stress/Psychological Distress 

Across all three studies, stress was substantial and clustered around academic and training demands. Almabadi et al. identified academic overload, fear of failure, limited time, and examination or clinical requirements as prominent stressors [13]. Punj et al. found that more than one-third of prosthodontic residents crossed the predefined high-stress threshold (modified GDES≥76), and year of study emerged as a significant risk factor. The most frequently endorsed stressors were lack of time for leisure, inadequate clinic staffing, and neglect of personal life; common coping themes were exercise, socializing, family time, self-care, time management, and connecting with others [14]. Inglehart et al. reported that prosthodontics residents had higher mean stress scores than pediatric dentistry and oral and maxillofacial surgery residents across several domains, including personal-life stress (3.67), faculty-related stress (3.66), lack-of-confidence-at-work stress (3.31), academic stress (3.24), and future-related stress (3.60) [15].

Burnout

Although occupational burnout was a prespecified review outcome, no eligible study used a validated burnout inventory. Consequently, no prosthodontics-specific burnout prevalence or severity estimate could be synthesized. The current evidence base, therefore, documents stress but not burnout in prosthodontic training.

Risk of Bias Assessment

**Figure 2:**
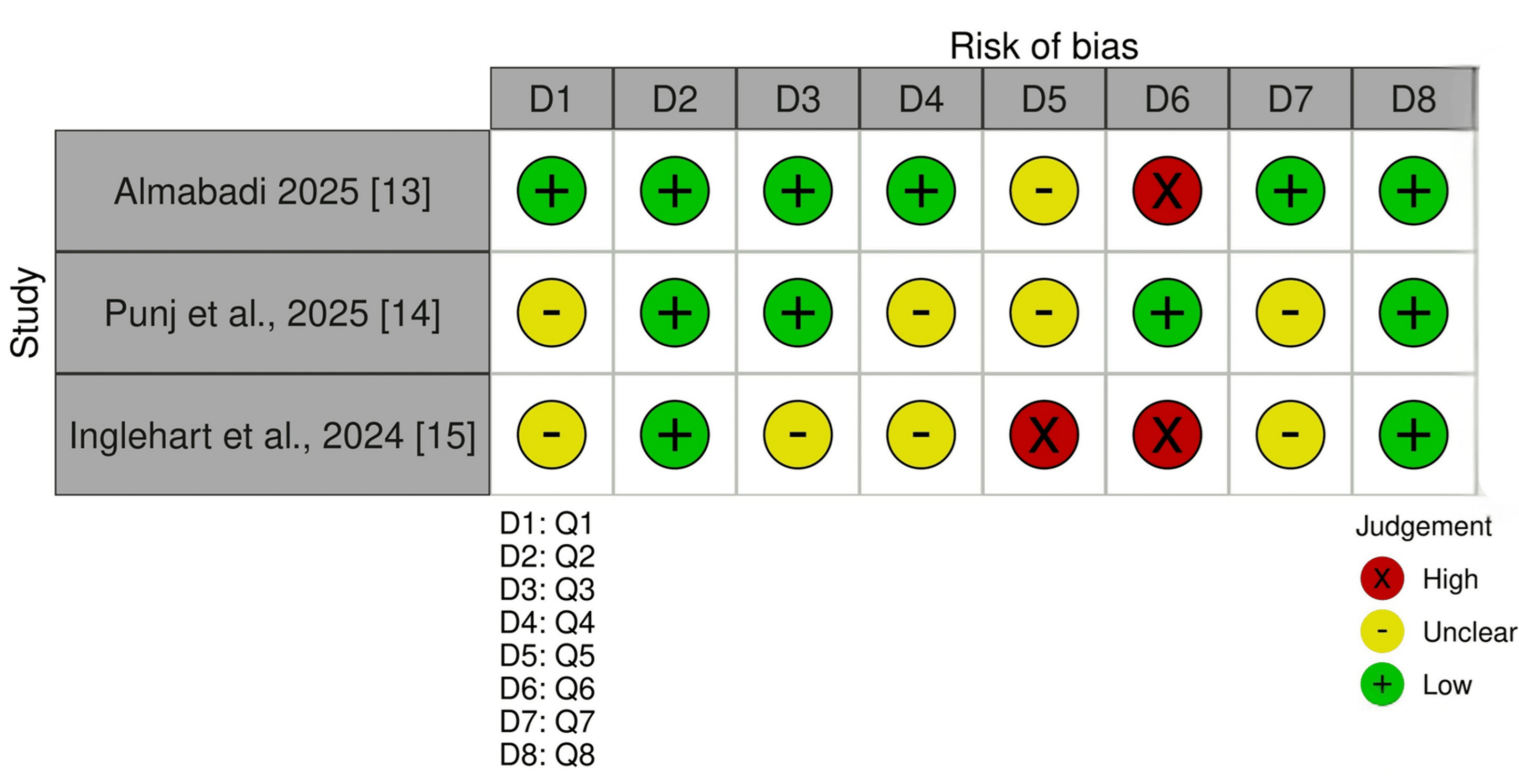
Item-level JBI traffic-light summary for included cross-sectional studies JBI: Joanna Briggs Institute

Methodological quality varied. Almabadi et al. met 6 of 8 JBI items (75%) with one unclear item and one identified limitation, mainly failure to address confounding [13]. Punj et al. met 4/8 items (50%), with four unclear judgements, reflecting uncertainty related to sampling and analytical reporting [14]. Inglehart et al. met 2/8 items (25%) and had the greatest concerns, particularly around confounding and selection-related issues [15]. Across the evidence base, the main threats to confidence were cross-sectional design, low or uneven response rates, limited handling of potential confounders, and sampling frameworks that may not fully represent the wider prosthodontic resident population. Figure 2 presents the item-level JBI traffic-light summary.

Across studies, the main methodological concerns were inadequate treatment of confounding, possible sampling bias, and response-rate limitations. These issues reduce confidence in the absolute magnitude of reported stress, although the direction of the findings was broadly consistent.

Meta-analysis was not performed because only three studies were eligible and because substantial clinical and methodological heterogeneity across populations and stress measurement tools precluded meaningful pooling. Therefore, results are presented as a narrative synthesis. Publication bias was not assessed because fewer than 10 studies were available.

Discussion

Methodology Justification

This review was undertaken to address an important evidence gap regarding prosthodontics-specific occupational strain, particularly within residency and postgraduate training, where complex rehabilitation planning, intensive clinical-laboratory coordination, time pressure, and high patient expectations may amplify psychological burden. Across the three eligible cross-sectional studies, prosthodontic residents demonstrated clinically relevant stress exposure, including comparatively higher mean stress-domain scores than other dental specialty residents in a multi-specialty cohort, a substantial proportion meeting a predefined threshold for high stress in a North American prosthodontic resident sample, and prominent stressors related to academic overload, fear of failure, time limitations, and meeting examination and clinical requirements in a Middle Eastern postgraduate prosthodontics cohort [13-15]. Collectively, these findings support the need to characterize measurement approaches and summarize stress- and burnout-related outcomes within prosthodontics training rather than extrapolating from general dentistry datasets.

These findings can be interpreted using established occupational stress frameworks. The Job Demands-Resources model proposes that sustained imbalance between high job demands and insufficient resources increases the risk of occupational strain and downstream burnout outcomes [8]. Prosthodontics training is plausibly high-demand because it combines prolonged technical procedures, longitudinal case responsibility, repeated treatment re-planning, and dependency on laboratory workflows. Burnout theory, as operationalized in the MBI, conceptualizes burnout across emotional exhaustion, depersonalization, and reduced personal accomplishment [7]. Importantly, perceived stress, psychological distress, and burnout are related but non-identical constructs. Burnout instruments such as the MBI and the CBI quantify burnout domains, whereas global stress tools such as the perceived stress scale (PSS) and symptom-oriented measures such as the depression anxiety stress scales (DASS) primarily assess perceived stress severity or stress symptom burden rather than burnout as a syndrome [7-11]. In the included prosthodontics studies, stress was measured using specialty- or training-oriented stress surveys rather than validated burnout inventories, which limits comparability with dentistry-wide burnout prevalence estimates and precludes any synthesis of burnout prevalence within prosthodontics cohorts [7,9,13-15].

Supporting and Contrasting Literature

The present findings are directionally consistent with the broader dentistry literature, which shows that psychological distress and burnout-related symptomatology are substantial among dental professionals [1,4,5]. Dentistry-wide evidence has linked occupational strain to high workload, limited recovery time, financial and staffing pressures, and other adverse work-related determinants [4,5,16-18]. In a large Chinese survey of dental medical staff using validated instruments, psychological distress was reported in 23.8% overall, with higher prevalence among dentists than dental nurses; factors associated with distress in dentists included lower income, burnout, high job stress, career-choice regret, and insufficient personal time [19]. Related evidence from the COVID-era dentistry has shown that infection concern, PPE-related burden, and income disruption may compound pre-existing occupational pressures [20-22]. Although these studies are not prosthodontics-specific, they strengthen the contextual argument that dental professionals frequently operate under demand-resource imbalance and that prosthodontics trainees likely experience these pressures within an especially demanding specialty environment.

A prosthodontics-specific explanation for the observed stress signal is plausible. Compared with some other dental specialties, prosthodontics requires high levels of technical precision and aesthetically driven outcomes, often with long treatment cycles and repeated patient engagement. These features may intensify performance pressure and increase the perceived consequences of error. The stressor patterns identified in the included studies, particularly faculty-related stress, personal-life constraints, academic overload, and examination or clinical requirements, are consistent with this specialty context [13-15]. At the same time, heterogeneity across instruments, thresholds, and survey contexts indicates that reported stress levels are sensitive to measurement choice, thereby limiting comparability and reinforcing the need for standardized assessment methods [23,24].

Strengths and Limitations

A strength of this review is its prosthodontics-focused scope and the use of structured critical appraisal, which together provide an evidence map directly relevant to specialty training rather than inferred from broader dental cohorts. However, several limitations should be considered. First, only three eligible studies were identified, limiting the breadth of inference. Second, all included studies were cross-sectional, which precludes causal interpretation. Third, substantial variation in outcome operationalization prevented quantitative pooling. Fourth, generalizability is restricted by single-country or single-institution sampling in some studies and by potential nonresponse effects, particularly where response rates were modest [13-15]. Finally, although burnout was a prespecified outcome, no eligible prosthodontics-specific studies used validated burnout inventories such as the MBI or CBI, preventing any estimation of burnout prevalence in prosthodontists or prosthodontic residents [7,9].

Future Directions

The consistent stress signal across countries supports proactive approaches within prosthodontic training programs and future research. Multicenter studies across different training systems should include both residents and practicing prosthodontists and should incorporate validated, standardized instruments for stress and burnout, such as the PSS, MBI, or CBI [7,9,10]. Future studies should also standardize reporting of plausible determinants, including workload, clinical requirements, staffing and laboratory access, supervisory climate, and recovery time, to enable subgroup analyses and intervention development. The systematic identification of an evidence gap in prosthodontics-specific burnout measurement is itself a key finding of this review and provides a clear agenda for generating reliable benchmarks and informing targeted educational and workplace interventions.

## Conclusions

Current evidence indicates that prosthodontics residents experience substantial perceived stress, with prominent stressors related to faculty interactions, academic workload, personal-life constraints, and meeting training requirements. However, a critical knowledge gap persists regarding occupational burnout in prosthodontics. No eligible studies used validated burnout inventories (e.g., MBI or CBI) in prosthodontists or prosthodontic residents, precluding any estimation of burnout prevalence. Consequently, while the signal for psychological distress is clear, the burden of burnout in this specialty remains unquantified. Future research should prioritize robust, multicenter studies using standardized metrics to accurately measure burnout and guide needed educational and workplace interventions.

## Appendices

Supplementary file 1

1. PubMed (MEDLINE)

Search strategy:(("Prosthodontics"[Mesh] OR "Prosthodontists"[Mesh] OR prosthodontic*[tiab] OR prosthodontist*[tiab] OR "prosthodontic resident*"[tiab] OR "prosthodontic trainee*"[tiab] OR "postgraduate prosthodontic*"[tiab]) AND ("Burnout, Professional"[Mesh] OR burnout[tiab] OR "professional burnout"[tiab] OR stress[tiab] OR "perceived stress"[tiab] OR "occupational stress"[tiab] OR "psychological distress"[tiab] OR exhaustion[tiab]))AND (english[lang])

2. Scopus

Search strategy: TITLE-ABS-KEY (prosthodontic* OR prosthodontist* OR "prosthodontic resident*" OR "prosthodontic trainee*" OR "postgraduate prosthodontic*") AND TITLE-ABS-KEY (burnout OR "professional burnout" OR stress OR "perceived stress" OR "occupational stress" OR "psychological distress" OR exhaustion) AND (LIMIT-TO (LANGUAGE, "English"))

3. Cochrane Library

Search strategy: (prosthodontic* OR prosthodontist* OR "prosthodontic resident*" OR "prosthodontic trainee*" OR "postgraduate prosthodontic*") AND (burnout OR "professional burnout" OR stress OR "perceived stress" OR "occupational stress" OR "psychological distress" OR exhaustion)

4. LILACS

Search strategy: (prosthodontic* OR prosthodontist* OR "prosthodontic resident*" OR "prosthodontic trainee*") AND (burnout OR stress OR "perceived stress" OR "occupational stress" OR "psychological distress")

5. Google Scholar

Primary search query: (prosthodontic OR prosthodontist OR "prosthodontic resident")(stress OR "perceived stress" OR "psychological distress" OR burnout OR "professional burnout")

Secondary focused query: ("prosthodontic resident" OR "postgraduate prosthodontics") ("Dental Environment Stress" OR DESS OR "Graduate Dental Environment Stress")

6. Shodhaganga (Theses Repository)

Keywords used: prosthodontics stress, prosthodontic residents stress, prosthodontics burnout, and prosthodontist burnout

Additional Search Methods

Reference list screening (backward citation searching) of included studies

Cross-checking of related articles during full-text assessment

Supplementary file 2

**Table 2:** Excluded studies

Author (year)	Full citation (Vancouver style)	Reason for exclusion	Explanation
Asiri A (2022)	Asiri A. Saudi Arabian students in postgraduate dental programs: investigating factors associated with burnout. F1000Research. 2022;11:705. doi:10.12688/f1000research.111000.2	Population not separable	Included multiple dental specialties (including prosthodontics), but burnout outcomes were not reported separately for prosthodontic residents; prosthodontic-specific data not extractable
Shetty A, et al. (2015)	Shetty A, Shetty A, Hegde MN, et al. Stress and burnout assessment among postgraduate dental students. Nitte Univ J Health Sci. 2015;5(1):31-35. doi:10.1055/s-0040-1703859	Population not separable	Multi-department postgraduate dental sample including prosthodontics; results presented overall and stratified by age/gender/year, not by specialty; prosthodontic data not separable
Divaris K, et al. (2012)	Divaris K, Lai CS, Polychronopoulou A, et al. Stress and burnout among Swiss dental residents. Schweiz Monatsschr Zahnmed. 2012;122(7-8):610-615. PMID:22914975	Population not separable	Included residents from multiple dental programs (including prosthodontics); outcomes not stratified by specialty/program type; prosthodontic-specific results not extractable
